# Multiple Dyselectrolytemia in a Chronic Alcohol Abuser: A Case Report

**DOI:** 10.7759/cureus.36389

**Published:** 2023-03-20

**Authors:** Dakshin Meenashi Sundaram, Vijaya Prakash Madesh, Donthireddy Rambrahma Reddy, Krishna Baliga

**Affiliations:** 1 Internal Medicine, Employees' State Insurance Corporation (ESIC) Medical College and Post Graduate Institute of Medical Science and Research (PGIMSR), Chennai, IND; 2 Nephrology, Employees' State Insurance Corporation (ESIC) Medical College and Post Graduate Institute of Medical Science and Research (PGIMSR), Chennai, IND

**Keywords:** hypomagnesemia, hypophosphatemia, hypocalcemia, other causes of hypokalemia, renal tubular dysfunction, alcohol abuse, dyselectrolytemia

## Abstract

Electrolyte disorders in alcohol-dependent patients can be due to a multitude of reasons. We discuss a patient with diabetes mellitus, seizure disorder, and alcoholism who presented with seizure episodes and vomiting following a binge alcohol intake. The evaluation showed life-threatening metabolic derangements that included hyponatremia, hypokalemia, hypomagnesemia, hypocalcemia, hypochloremia, hypophosphatemia with elevated blood glucose, and metabolic alkalosis with a normal anion gap. Subsequently, a detailed urinary analysis revealed a urinary loss of electrolytes. We emphasize that alcohol-induced tubular injury is a possibility when such a clinical presentation is seen in the emergency room. The complex interplay of various electrolytes in homeostasis posed a great challenge in the management of this patient. Our case reiterates this intricate electrolyte correction policy.

## Introduction

Electrolyte abnormalities are a common occurrence in patients with chronic alcohol use disorders. The clinical significance of these abnormalities depends on the amount and duration of use [1]. Electrolyte disorders in alcohol-dependent patients can be due to a multitude of reasons [1]. Beer-potomania-profound hyponatremia has been widely described in the literature [2]. But multiple dyselectrolytemia in chronic alcohol abusers have not been frequently described. Prioritizing the correction of certain electrolytes over others is a crucial life-saving strategy in such cases, keeping in mind the comorbid conditions and the influence of one electrolyte over the others. Our case reiterates this intricate electrolyte correction policy.

## Case presentation

A 42-year-old male presented to the emergency department with a history of seizures, followed by an episode of vomiting and bilateral lower limb weakness of one-day duration following a binge alcohol intake five days ago. He had been a known diabetic for 10 years, had a seizure disorder for one year, and was noncompliant with medications. He consumed varying quantities of alcohol almost daily for more than 15 years. On examination, he was conscious, disoriented [Glasgow Coma Scale of Eye-Opening (E) 4, Verbal (V) 4, and Motor (M) 6], and dehydrated. Motor system examination of the lower limbs revealed hypotonic flaccid paralysis of both lower limbs with a power of 2/5; areflexia; bilateral plantar flexors. Motor system examination of the upper limb was normal. Bowel and bladder functions were normal. He had a heart rate of 85/min and blood pressure of 130/80 mm hg. Capillary blood glucose was high. The computed tomography of the brain was normal. His initial laboratory evaluations are listed in Table 1.

**Table 1:** Initial laboratory parameters

Parameter	Observed value	Normal range
Haemoglobin	10.5 g/dL	13.3–17.2 g/dL
WBC	10,620 cells	3700–9700 cells/mm^3^
Random blood sugar	652 mg/dL	70–140 mg/dl
Urine ketones	Negative	Negative
pH	7.48	7.35–7.45
pO_2_	83 mmHg	83–108 mmHg
pCO_2_	51.3 mmHg	32–48 mmHg
$\textrm{HCO}_{3}^{-}$	35.7 mmol/L	23–29 mmol/L
Serum sodium	129 mmol/L	135–148 mmol/L
Serum potassium	1.55 mmol/L	3.50–5.10 mmol/L
Serum chloride	92 mmol/L	98–106 mmol/L
Serum magnesium	1.5 mg/dL	1.6–2.6 mg/dL
Serum phosphate	0.8 mg/dL	2.5–4.5 mg/dL
Serum calcium	6.14 mg/dL	8.80–11 mg/dL
Corrected calcium	6.6 mg/dL	
Creatine phosphokinase	1896 U/L	55–170 U/L
Lactate dehydrogenase	431 U/L	124–246 U/L
Serum osmolarity	311 mosm/kg	275–300 mosm/kg
Urine osmolarity	307 mosm/kg	900–1200 mosm/Kg
Serum creatinine	1.32 mg/dL	0.22–1.07 mg/dL
Serum urea	40 mg/dL	5.00–40.00 mg/dL
Serum parathormone	55 pg/mL	10–55 pg/mL
Serum vitamin D3	38.4 ng/mL	20–40 ng/mL
Serum protein	6.70 g/dL	6.30–8.20 g/dL
Serum albumin	3.40 g/dL	3.50–5.00 g/dL

He was worked up for multiple dyselectrolytemia. Thyroid and cortisol levels were normal. He had severe hypokalemia, causing rhabdomyolysis and limb weakness. His 24-hour urinary potassium level was 57.4 mmol/day (normally <15 mmol/day), and his urine potassium to creatinine ratio was 17 (normally <13), suggesting renal loss of potassium. Twenty-four-hour urinary magnesium was 53 mg/day, and fractional excretion of magnesium was 10%, both indicating renal loss of magnesium. Severe hypomagnesemia also contributed to severe hypokalemia. Hypocalcemia was attributed to urinary loss of calcium, confirmed by a urine spot calcium to creatinine ratio of 0.317 (normal <0.14), along with hypomagnesemia. Vitamin D and parathyroid hormone levels were normal.

Central venous access was placed, and intravenous potassium and phosphorus corrections were prioritized and initiated immediately. Oral formulations of potassium chloride were also added. 0.9% normal saline solution was chosen for correction in view of hyponatremia. Intravenous magnesium and calcium corrections were initiated simultaneously alongside oral calcium supplements. Urine output was meticulously monitored. He was given 120 mEq of potassium chloride, 15 ml of potassium phosphate (45 mM of phosphorus and 66 mEq of potassium), 3 g of calcium gluconate, and 2 g of magnesium sulfate per day in divided doses on the day of admission. In view of his severe hypokalemia, insulin was initially withheld for the management of his blood glucose. On day 2 of admission, his lower-limb weakness improved to power 4/5. Blood glucose was uncontrolled, and hence subcutaneous insulin was started cautiously along with potassium supplementation. His electrolyte abnormalities were corrected as mentioned above, and they slowly improved over the next few days (Table 2).

**Table 2:** Serial electrolyte values

	On admission	Day 1	Day 3	Day 5	Day 7
Sodium (mmol/L)	129	135	141	136	133
Potassium (mmol/L)	1.55	1.76	2.03	2.42	3.40
Magnesium (mg/dL)	1.5	1.5	0.9	1	1.8
Phosphorus (mg/dL)	0.8	1.6	2.3	2.1	2.0
Calcium (mg/dL)	6.14	5.07	5.83	6.0	6.18
Glucose (mg/dL)	627	520	205	140	162

In a week’s time, his electrolyte derangements were corrected except for mild hypokalemia and hypocalcemia. His blood glucose was controlled. He was walking independently without any residual weakness. After symptomatic improvement, the patient was discharged against medical advice, and he was lost to follow-up.

## Discussion

Metabolic disturbances are not uncommon in people with chronic alcohol abuse [1]. Alcohol has a direct role in pathophysiological derangements, and other contributory factors like malnutrition and intercurrent illnesses can also induce these disorders. The clinical presentation of these electrolyte disturbances may be too nonspecific and can delay the diagnosis. This patient’s presentation with a seizure episode raised the possibility of a breakthrough seizure or an alcohol withdrawal seizure. He was screened for possible metabolic disturbances, which revealed multiple life-threatening dyselectrolytemias. The quantity and chronicity of alcohol consumption influence the clinical significance of these electrolyte imbalances [1].

Hypokalemia is seen in about 50% of patients hospitalized for chronic alcohol use disorder [1]. Causes of hypokalemia in alcoholics could include inadequate intake, urinary loss due to coupling of increased distal sodium delivery and increased aldosterone level, magnesium deficiency, diarrhea, cellular shift due to insulin release, correction of acidosis, respiratory alkalosis, and β2-adrenergic stimulation [1,2]. Profuse vomiting and underlying ketoacidosis cause increased urinary loss of potassium due to increased mineralocorticoid levels and increased delivery of sodium to the distal nephron (which is due to the non-resorbable anion effect of bicarbonate and keto acid salt) [2]. Coexistent magnesium deficiency also leads to inappropriate potassium loss, as decreased intracellular magnesium causes upregulation of renal outer medullary potassium (ROMK) channels on the apical membrane of the distal nephron and normally inhibits potassium secretion [2,3]. Clinical features of hypokalemia include cardiac arrhythmias, muscle paralysis, myopathy, ileus, and rhabdomyolysis. Our patient had features of rhabdomyolysis; renal loss of potassium along with magnesium deficiency contributed to hypokalemia [4]. Hypokalemia with concomitant hypomagnesemia as described above will be refractory to correction until the magnesium is corrected.

Hypomagnesemia is seen in almost one-third of patients with chronic alcohol use disorder [5]. Hypomagnesemia in chronic alcoholics can be of nutritional origin, secondary to phosphate deficiency, or due to tubular injury by alcohol. It can cause neuromuscular irritability in the form of seizures, tremors, and limb weakness. Our patient had seizures and limb weakness, a renal loss of magnesium, and a phosphate deficiency causing severe hypomagnesemia. Hypomagnesaemia is the most common electrolyte disturbance in alcoholics [4,5]. Magnesium deficiency causes secondary potassium depletion [4,6].

Acute hypophosphatemia usually develops in up to 50% of patients over the first two to three days after hospitalization for problems related to chronic alcohol overuse. Hypophosphatemia in alcoholics can be due to nutrition, increased renal loss, use of antacids, chronic diarrhea, vomiting, and magnesium deficiency. Despite having a nutritional cause for low phosphate, excretion of urinary phosphate is increased because of renal tubular dysfunction [7]. Tubular abnormalities may be due to the dysfunction of apically located transporters and to decreased activity of the sodium-potassium ATPase, both of which are related to structural changes in the phospholipid bilayer of the cell membrane. Clinical manifestations include cardiac dysfunction, skeletal muscle weakness, and hemolysis. Hypophosphatemia in our patient can also be due to an underlying magnesium deficiency. The urine phosphorus level was not tested for our patient. Generalized tubular dysfunction, induced by ethanol, which decreases the renal threshold for phosphate excretion, is a possibility [8]. Hypokalemia and hypophosphatemia can cause rhabdomyolysis [9].

Hypocalcemia in chronic alcohol abusers can be due to the direct effect of alcohol, which causes urinary calcium loss [10]. Alcohol also decreases the activity of Na+ and K+-ATPase in the renal proximal convoluted tubule cells, causing a decrease in the tubular reabsorption of calcium, which could have been worsened by concomitant hypomagnesemia. Another possible mechanism that links hypocalcemia with hypomagnesemia is the peripheral resistance of parathyroid hormone secondary to hypomagnesemia, which prevents the normal physiologic maintenance of serum calcium levels. Alcohol is also postulated to cause hypocalcemia secondary to poor intestinal absorption owing to its direct effect on vitamin D metabolism.

Possible acid-base disturbances in alcoholics include alcoholic ketoacidosis, lactic acidosis, metabolic alkalosis, and respiratory alkalosis [1]. Most frequently, we encounter respiratory acidosis and metabolic acidosis [7]. Our patient had metabolic alkalosis, possibly due to vomiting and hypokalemia.

Hyponatremia in alcoholics can be due to various causes like beer potomania, hyperglycemia-induced extracellular free water shift, and continuing free water intake, along with enhanced anti-diuretic hormone (ADH) secretion in the presence of volume depletion [3]. Hyponatremia in our patient is possibly secondary to hyperglycemia and thus is not a true hyponatremia.

Ethanol-induced tubular dysfunction is one probable cause for these metabolic abnormalities. Direct effects on the tubular cell membranes can cause structural damage to the phospholipid bilayers. In the tubules, alcohol also induces mitochondrial toxicity with a decrease in ATP production through oxidative stress. This further aggravates tubular dysfunction, thereby affecting the action of transporters. However, tubular dysfunction usually improves within days of abstinence from alcohol [10].

**Figure 1:**
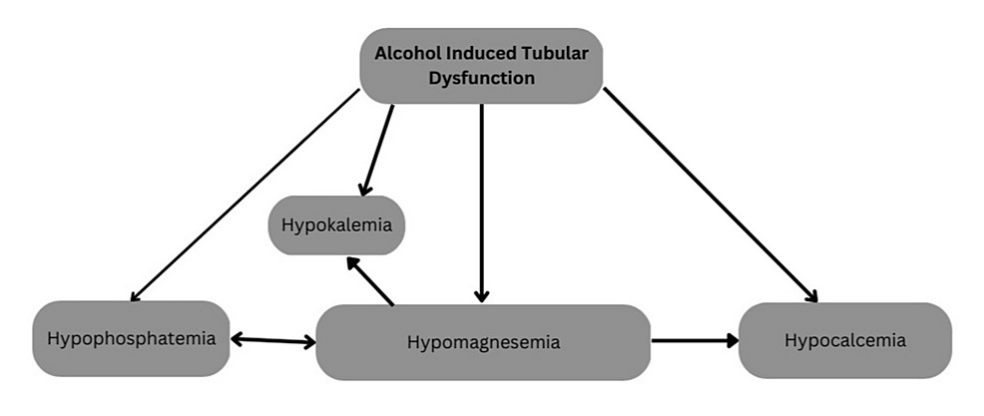
Complex interplay of electrolytes

The management of multiple metabolic abnormalities poses a great challenge because of the multiple influences of one electrolyte over the other (Figure 1).

Prioritizing which electrolyte to correct first also plays a major role in avoiding potential complications. In such a scenario as our patients, we prioritized potassium and phosphate correction at first, as they have a direct effect on the heart and can lead to arrhythmias and cardiac dysfunction. It was followed by magnesium correction, as it is necessary for replenishing potassium and phosphate levels [6,11,12]. Normal saline, which was used to give the electrolyte corrections, corrected the underlying false hyponatremia. Hypertonic saline was not administered to obviate overcorrection of the plasma osmolality.

## Conclusions

Electrolyte imbalances can have varied presentations and can result in life-threatening complications when treatment is delayed. Prompt identification will help in the immediate initiation of correction. Potassium correction prior to blood sugar management is vital to prevent life-threatening hypokalemia in such a case. The complex interplay of electrolytes with other metabolic disturbances, especially chronic alcohol abuse, poses a great challenge to the physician, both in diagnosis and treatment. Ethanol-induced tubular dysfunction should be borne in mind while dealing with such patients. Physiologic priorities of correction have to be planned and carried out accordingly.
